# Structures of α-galactosaminidases from the CAZy GH114 family and homologs defining a new GH191 family of glycosidases

**DOI:** 10.1107/S2059798325002864

**Published:** 2025-04-15

**Authors:** Christian Roth, Olga V. Moroz, Suzan A. D. Miranda, Lucas Jahn, Elena V. Blagova, Andrey A. Lebedev, Dorotea R. Segura, Mary A. Stringer, Esben P. Friis, João P. L. Franco Cairo, Gideon J. Davies, Keith S. Wilson

**Affiliations:** a Department for Biomolecular Systems, Carbohydrates Structure and Function Max Planck Institute of Colloids and Interfaces Arnimallee 22 14195Berlin Germany; b York Structural Biology Laboratory, Department of Chemistry University of York YorkYO10 5DD United Kingdom; c CCP4 STFC Rutherford Appleton Laboratory Harwell Oxford DidcotOX11 0QX United Kingdom; d Novonesis A/S, Biologiens Vej 2, 2800Kgs Lyngby, Denmark; University of Cambridge, United Kingdom

**Keywords:** galactosaminidases, structure, glycoside hydrolase family, crystal pathology

## Abstract

Endo-galactosaminidases are an underexplored family of enzymes involved in the degradation of galactosaminogalactan (GAG) and other galactosamine-containing cationic exopolysaccharides produced by fungi and bacteria. These exopolysaccharides are part of the cell wall and extracellular matrix of microbial communities. Currently, these galactosaminidases are found in three distinct CAZy families: GH114, GH135 and GH166. Despite the widespread occurrence of these enzymes in nearly all bacterial and fungal clades, only limited biochemical and structural data are available for these three groups. To expand our knowledge of endo-galactosaminidases, we selected several sequences predicted to encode endo-galactosaminidases and produced them recombinantly for structural and functional studies. Only very few predicted proteins could be produced in soluble form, and activity against bacterial Pel (pellicle) polysaccharide could only be confirmed for one enzyme. Here, we report the structures of two bacterial and one fungal enzyme. Whereas the fungal enzyme belongs to family GH114, the two bacterial enzymes do not lie in the current GH families but instead define a new family, GH191. During structure solution we realized that crystals of all three enzymes had various defects including twinning and partial disorder, which in the case of a more severe pathology in one of the structures required the design of a specialized refinement/model-building protocol. Comparison of the structures revealed several features that might be responsible for the described activity pattern and substrate specificity compared with other GAG-degrading enzymes.

## Abstract 2

Structural and functional analysis of a new fungal GH114 endo-α-1,4-galactosaminidase, along with homologs that establish a new GH191 family of glycoside hydrolases, are reported. In addition, various crystal pathologies led to the development of a new refinement procedure for disordered structures.

## 1. Introduction

Positively charged polysaccharides are important components of the cell wall and extracellular matrix of many microorganisms. Galactosaminogalactan (GAG) is a positively charged extracellular polysaccharide composed of varying amounts of galactose (Gal), *N*-acetylgalactosamine (GalNAc) and galactosamine (GalN). It is part of the exopolysaccharide components of the cell wall and extracellular matrix of various fungal species. A similar cationic polysaccharide containing GalNAc and GalN, but no Gal, is found in the extracellular polymeric substance matrix (EPS) of *Pseudomonas aeruginosa*, where it is termed the pellicle (Pel) polysaccharide (Baker *et al.*, 2016 ▸; Colvin *et al.*, 2013 ▸; Flemming & Wingender, 2010 ▸; Flemming *et al.*, 2016 ▸; Mitchell *et al.*, 2016*a* ▸,*b* ▸; Gow *et al.*, 2017 ▸; Le Mauff *et al.*, 2022 ▸). In both bacteria and fungi the production of these polysaccharides is correlated with the pathogenicity (Brown *et al.*, 2012 ▸; Speth *et al.*, 2019 ▸). Specific aspergilli, for example *Aspergillus fumigatus* or *A. parasiticus*, produce high amounts of GAG as part of their cell wall and extracellular matrix, shielding the fungus against the immune system (Takada *et al.*, 1981 ▸; Briard *et al.*, 2016 ▸). Thus, GAGs are one of the reasons for the persistence of fungal infections (Brown *et al.*, 2012 ▸; Speth *et al.*, 2019 ▸; Gravelat *et al.*, 2013 ▸). Given the critical role of these cationic polysaccharides in microbial pathogenicity and their structural diversity, the study of new glycoside hydrolases capable of degrading GAGs can provide insights into their biological functions and offer novel therapeutic strategies to combat persistent infections.

Counterintuitively, GAG-degrading glycosidases play an important role in biosynthesis. For example, the synthesis and maturation pathway of *A. fumigatus* GAG has been studied in detail (Gow *et al.*, 2017 ▸; Gravelat *et al.*, 2013 ▸; Low & Howell, 2018 ▸; Bamford *et al.*, 2015 ▸). In brief, a carbohydrate isomerase (Uge3) produces the intracellular building blocks from UDP-Glc for UDP-Gal and from UDP-GlcNAc for UDP-GalNAc, which are then used by a transmembrane GAG synthase that assembles the polymer (Gow *et al.*, 2017 ▸). An extracellular deacetylase (Agd3) is employed to partially deacetylate stretches of GalNAc monomers to produce GalN, which leads to an overall positive charge, a hallmark of this polysaccharide (Bamford *et al.*, 2020 ▸). Two membrane-bound glycoside hydrolases are necessary for the post-synthetic modification and remodelling of the extracellular polygalactosamine, namely Ega3 and Sph3 (Bamford *et al.*, 2015 ▸, 2019 ▸). Knockout mutants of *A. fumigatus* strains lacking Sph3 demonstrated that Sph3 is essential for GAG biosynthesis. (Bamford *et al.*, 2015 ▸). Sph3 is most probably in close proximity to the integral membrane glycosyltransferase Gtb3 and cleaves the polymer when it leaves the extracellular site of Gtb3, probably to prevent clogging (Bamford *et al.*, 2015 ▸). A detailed analysis of Sph3 revealed that the enzyme consists of a (β/α)_8_ TIM barrel with a specificity for fully acetylated GAG, consistent with its proposed association with Gtb3. Due to the low sequence similarity to known CAZy families, together with the functional data, a new family 135 within the CAZy classification scheme was created (Bamford *et al.*, 2015 ▸; Drula *et al.*, 2022 ▸).

The second glycoside hydrolase, Ega3, was assigned to CAZy family GH114 and shows a high specificity for deacetylated galactosamine stretches within the GAG polymer (Bamford *et al.*, 2019 ▸). The structure of Ega3 (PDB entries 6oj1 and 6ojb) revealed a (β/α)_8_ barrel with a deep, strongly electronegative binding cleft, which stretches over the whole surface (Bamford *et al.*, 2019 ▸). The active-site residues have been identified based on mutagenesis studies and are structurally arranged consistent with the proposed retaining mechanism (Bamford *et al.*, 2019 ▸). A galactosamine monomer was observed within this cleft in the crystal structure, providing some insight as to how the enzyme might bind its substrate (Bamford *et al.*, 2019 ▸).

In bacteria, the production of the pellicle (Pel) polysaccharide involves the *pel* gene cluster consisting of seven open reading frames. Similar to the fungal system, an enzyme production and modification system is necessary. PelA is a glycoside hydrolase/deacetylase that is required for the synthesis of Pel (Colvin *et al.*, 2013 ▸). The hydrolase domain of PelA cleaves within partially deacylated regions of the polymer and is the founding member of GH family 166 (Le Mauff *et al.*, 2019 ▸). Although fungi are mostly known to produce GAG, and the synthesis and modification pathway is best understood for *A. fumigatus*, the majority of the enzymes in all three CaZy families are of bacterial origin. To date, only a few enzymes from each family have been characterized in detail, with GH114 and GH135 having fungal members (Ega3 and Sph3, respectively) and one bacterial member of GH114, with *Pseudomonas putida* sp. 881 (*Pp*GH114) having been partially characterized based on an an enzyme preparation purified from the native source (Tamura *et al.*, 1988 ▸, 1992 ▸). Another enzyme, from *Streptomyces griseus*, has been shown to have galactosaminidase activity, but no sequence is available and therefore assignment of a CAZy family is not possible (Reissig *et al.*, 1975 ▸). For GH166, only the founding member from *P. aeruginosa* has been characterized. Notably, all three enzyme families share structural similarities. All characterized enzymes in the three families are endo-type enzymes, share a conserved (β/α)_8_ TIM-barrel domain and follow a retaining mechanism with conserved active-site residues. The partially characterized *Pp*GH114 was also shown to be an endo-type hydrolase, following a retaining mechanism, with a minimal substrate size of four monomers (Tamura *et al.*, 1992 ▸). This enzyme has weak transglycosylation activity, further supporting a retaining mechanism (Tamura *et al.*, 1992 ▸). A more detailed analysis of the active-site geometry, the differences in specificity and the exact catalytic itinerary is still lacking due to the lack of functional and crucially structural data for other members of the families, in particular structures in which the substrate or substrate analogue are missing.

Given the importance of polysaccharide degradation in these systems, we set out to investigate members of the endo-galactosaminidase superfamily as potential tools to interrogate the composition of the extracellular matrix of bacteria and fungi. Furthermore, we want to better understand the structural and functional relationship between the different CAZy families. Here, we describe structure–function investigations of two bacterial enzymes: an enzyme from *Thermotoga maritima* previously suggested to be a galactosaminidase and another from an environmental sample (termed Env-GH191 here), which has 99.2% identity to an enzyme from *Myxococcus fulvus* with NCBI reference WP_074950922.1 (Naumoff & Stepuschenko, 2011 ▸). We also studied a fungal homolog from *Fusarium solani*, a pathogenic fungus that is notable as the causative agent of keratitis (inflammation of the cornea; Zhang *et al.*, 2006 ▸). Our results extend the to date very limited structural and functional data on endo-galactosaminidases, providing a significant advance towards understanding the biological role of these enzymes and providing a structural platform for their industrial application. Our analyses define a new CAZy family, GH191, of endo-galactosaminidases, distinct from the established families GH114, GH135 and GH166. Furthermore, we define the structural similarities and differences between the families and may help to further elucidate the evolutionary relationship and functional role of these enzymes. In addition, the procedure used for refinement and model building for the Env-GH191 crystal structure, which had partial lattice disorder, is described in detail and can be used for similar cases of severe crystal pathology.

## 2. Materials and methods

### 2.1. Phylogenetic analysis of putative endo-galactosaminidase sequences

The sequences were selected from the Carbohydrate-Active enZYme database (CAZy; Drula *et al.*, 2022 ▸), ensuring that the sequences correspond to a subset of bacteria that are accessible via the German Collection of Microorganism and Cell Cultures GmbH (DSMZ; https://www.dsmz.de). The sequence of *Tm*GH191 was obtained from the European Nucleotide Archive (ENA), whereas the sequence of Env-GH191 was provided by Novonesis. The sequences were analysed and the phylogenetic tree was subsequently visualized via the *MEGA* software suite (Tamura *et al.*, 2021 ▸).

### 2.2. Cloning, gene expression, protein production and purification

#### 2.2.1. Putative bacterial GH114 enzymes

All gene sequences, except for that from *Pseudomonas putida*, were obtained by amplification from the genomic DNA of the respective bacteria: *Burkholderia cepacia* DSM7288, *Cupriavidus necator* H16 DSM428, *Nocardia* sp. Root240 DSM102477, *Aeromicrobium* sp. Root495 DSM102310 and *Streptomyces platensis* DSM40041. The respective strains were grown in Soy broth medium to obtain sufficient cell material for the preparation of genomic DNA. The DNA was prepped using the GeneJet genomic DNA-preparation kit from Sigma–Aldrich. The mature *P. putida* sequence was obtained by gene synthesis in pUC19 from GenScript. Subsequently, the coding sequence of each putative mature GH114 endo-galactosaminidase was amplified and cloned in either pET-28b (Agilent), pET-YSBL3CLIC (Fogg & Wilkinson, 2008 ▸) and/or pET-MBP1b (Agilent) by Gibson assembly. The correct insertion of the sequences was confirmed by sequencing. The correct plasmids were transformed in *Escherichia coli* Bl21(DE3) Gold (Agilent) and *E. coli* Bl21(DE3) Rosetta (Agilent) cells to test for soluble protein production.

#### 2.2.2. GH191 family endo-galactosaminidases

##### 2.2.2.1. *Tm*GH191

*Tm*GH191 was cloned from the genomic DNA of *T. maritima* DSM 3109 obtained from the DSMZ. The gene without the signal peptide, coding for amino acids 27–323, was cloned into the pET-YSBL3CLIC vector (Fogg & Wilkinson, 2008 ▸). One colony of the bacterial strain containing the vector with the gene of interest was used to inoculate lysogeny broth (LB) medium containing kanamycin at a concentration of 50 µg ml^−1^ and incubated at 37°C and 250 rev min^−1^ for 16 h. One litre of Terrific Broth (TB) medium with the appropriate antibiotic was inoculated with the LB culture at a dilution of 1:100. The culture was incubated at 37° and 220 rev min^−1^. When an OD of 1.2 was reached, protein expression was induced by adding isopropyl β-d-1-galactopyranoside (IPTG) to a final concentration of 0.1 m*M* and the culture was incubated at 16°C and 220 rev min^−1^ for a further 16 h. The bacteria were harvested by centrifugation at 4500*g* for 25 min. The supernatant was discarded and the pellet was resuspended in His-binding buffer (50 m*M* Tris pH 8.0, 500 m*M* NaCl, 10 m*M* imidazole, 1 m*M* DTT) and centrifuged for a further 30 min at 4500*g*. The supernatant was discarded and the pellet was stored at −20°C.

The thawed cells were resuspended in His-binding buffer and lysed using an Emulsiflex C3 cell disruptor (Avestin) by passing the cells through the instrument twice at a pressure of 103 MPa. The lysed cell extract was centrifuged at 50 000*g* for 45 min. The supernatant was applied onto a 5 ml HisTrap column (Cytiva) using an NGC chromatography system from Bio-Rad. The protein was eluted using a linear gradient of imidazole to a final concentration of 500 m*M* over five column volumes (CV). Fractions containing *Tm*GH191 were combined, concentrated and applied onto a Superdex S200 16/600 column equilibrated with 10 m*M* Tris pH 7.0, 150 m*M* NaCl 1 m*M* DTT. Following this size-exclusion chromatography step the fractions containing *Tm*GH191 were combined, concentrated to 20 mg ml^−1^, aliquoted and stored at −80°C.

##### 2.2.2.2. Env-GH191

The DNA encoding the Env-GH191 gene was isolated from a metagenome sample collected from a drainwater domestic environment in Denmark and submitted to full genome sequencing using Illumina technology (Hu *et al.*, 2015 ▸). The genome sequence was analysed for protein sequences that had glycoside hydrolase domains (according to the CAZY definition). A sequence containing a Glyco_hydro_114 domain, as defined in PFAM (PF03537, Pfam version 31.0; Finn *et al.*, 2016 ▸), was identified in the genome (GenBank PP187390). It had 99% (100% for the catalytic domain) sequence identity to the endo-α-1,4-polygalactosaminidase from *M. fulvus* (NCBI reference WP_074950922.1), but it is annotated here as ‘Env’ for environmental sample because the exact host has not been experimentally identified.

The DNA encoding the mature peptide predicted by *SignalP* (Bendtsen *et al.*, 2004 ▸) was amplified from the genomic DNA by standard PCR techniques using specific primers containing an overhang to the cloning vector. The amplified PCR fragment was then inserted into a *Bacillus* expression plasmid as described previously, with an affinity-tag sequence to simplify the purification process (Moroz *et al.*, 2017 ▸). In brief, the DNA encoding the mature polypeptide was cloned with the In-Fusion HD EcoDry Cloning Kit in frame to the *Bacillus clausii* secretion signal, BcSP, which replaced the native secretion signal sequence, followed by a N-terminal polyhistidine tag (HHHHHHPR). One recombinant *B. subtilis* clone containing the integrated GH114 expression construct was selected and cultivated on a rotary shaking table in 500 ml baffled Erlenmeyer flasks containing 100 ml LB medium supplemented with 34 mg l^−1^ chloramphenicol. The culture was cultivated for three days at 30°C.

The supernatant was harvested by centrifuging the culture broth for 30 min at 15 000*g* and submitted to standard His-tag purification by immobilized metal chromatography (IMAC) using nickel as the metal ion on 5 ml HisTrap Excel columns (GE Healthcare Life Sciences) and eluting with imidazole. An extra purification step was carried out by size-exclusion chromatography (SEC) using a HiLoad 26/600 Superdex 75 pg column (Cytiva) equilibrated in 50 m*M* HEPES, 100 m*M* NaCl pH 7.5. SEC fractions with 35 kDa single-band purity, assessed by SDS–PAGE, were concentrated using 10 kDa molecular-weight cutoff Sartorius Vivaspin 20 centrifugal concentrators (Sartorius). The purity of the sample was checked by SDS–PAGE and the concentration was determined by absorbance at 280 nm after buffer exchange into 50 m*M* HEPES, 100 m*M* NaCl pH 7.0. Prior to crystallization, the sample was concentrated to 20 mg ml^−1^, aliquoted and stored at −80°C.

For crystallization, a construct corresponding to the catalytic domain (residues 197–468) only was expressed and purified in the same way as described for the full-length protein.

#### 2.2.3. GH114 from *Fusarium solani*

The gene encoding a putative endo-α-1,4-polygalactos­aminidase belonging to family GH114, as defined by CAZy, was cloned from a strain of *F. solani* that was isolated from an environmental sample collected in Denmark. Chromosomal DNA was isolated from the strain, and the whole-genome sequence was purchased from Exiqon A/S, Vedbaek, Denmark. The genome sequence was assembled with the *SPAdes Genome Assembler* (version 3.5.0) and annotated with the *GeneMark* version 2.3c gene-prediction software (Ter-Hovhannisyan *et al.*, 2008 ▸). Peptides predicted from the annotated genome were searched for similarity to the GH114 domain and *Fs*GH114 was identified. The corresponding DNA sequence was PCR-amplified from *F. solani* genomic DNA with gene-specific primers that also append a Kozak translation-initiation sequence, TCACC, immediately 5′ of the start codon and cloned into an *Aspergillus* expression vector. The cloned *Fs*GH114-encoding gene was sequenced and confirmed to be identical to the corresponding gene found in the genome sequence, and was transformed into *A. oryzae* (Christensen *et al.*, 1988 ▸). Transformants were selected during regeneration from protoplasts based on the ability, conferred by a selectable marker in the expression vector, to utilize acetamide as a nitrogen source, and were subsequently re-isolated under selection.

Production of recombinant *Fs*GH114 was evaluated by culturing the transformants in 96-well deep-well microtiter plates for four days at 30°C in 0.25 ml of both YPG medium and DAP-4C-1 medium and monitoring protein expression by SDS–PAGE. A single *Aspergillus* transformant was selected, and the transformants were cultured in 500 ml baffled flasks containing 150 ml DAP-4 C-1 medium. The cultures were shaken on a rotary table at 150 rev min^−1^ for four days. The culture broth was subsequently separated from cellular material by passage through a 0.22 µm filtration unit. The pH of the filtered sample was adjusted to around pH 7.5 and 1.8 *M* ammonium sulfate was added. The sample was applied onto a 5 ml HiTrap Phenyl (HS) column on an ÄKTAexplorer. Prior to loading, the column had been equilibrated within 5 CV of 50 m*M* HEPES, 1.8 *M* ammonium sulfate (AMS) pH 7. To remove unbound material, the column was washed with 5 CV of 50 m*M* HEPES, 1.8 *M* AMS pH 7. The target protein was eluted from the column into a 10 ml loop using 50 m*M* HEPES, 20% 2-propanol pH 7. From the loop, the sample was loaded onto a desalting column (HiPrep 26/10 Desalting) and eluted with 50 m*M* HEPES, 100 m*M* NaCl pH 7.0. As a final purification step, the desalted protein was applied onto a 1 ml HiTrap Blue HP column on an ÄKTApure system and eluted with 50 m*M* Tris, 1 *M* NaCI pH 8.0. Based on the chromatogram, relevant fractions were collected. Protein concentration in the final sample was estimated by measuring the absorption at 280 nm. The sample was further concentrated to 20 mg ml^−1^, aliquoted and stored at −80°C.

### 2.3. Functional characterization

#### 2.3.1. GAG purification

The soluble fractions of GAG oligomers were purified from the supernatant of *A. fumigatus* (CEA10) cultures as described previously (Lee *et al.*, 2016 ▸). Briefly, the culture supernatant was mixed with 2.5 volumes of ethanol to precipitate the galactosamine. The precipitate was separated by centrifugation at 10 000*g* for 1 h. The precipitate was dried, resuspended in water, centrifuged for a further 1 h at 10 000*g* to remove any insoluble particles and freeze-dried. The freeze-dried pellet was weighed and redissolved in water to a final concentration of 10 mg ml^−1^.

#### 2.3.2. Pel substrate extraction

A crude extract of extracellular polymeric substance (EPS) was prepared from *Pseudomonas aeruginosa* PA14 (DSM 19882) as follows. The strain was streaked on Tryptone Soya Agar (TSA; pH 7.3; Oxoid, Basingstoke, United Kingdom) and incubated for three days at 30°C. Flasks with T-broth [10 g l^−1^ Bacto Tryptone (BD), 5 g l^−1^ sodium chloride (Sigma–Aldrich)] were then inoculated with single colonies and incubated statically for six days at 20°C. The biomass formed on the broth surface was carefully collected, transferred to tubes and pelleted by centrifugation at 16 000*g* for 5 min at 25°C. The pellets were resuspended in 3 *M* NaCl, vortexed vigorously and incubated for 15 min at ambient temperature to extract the surface-associated polymer. The extracts were then re-pelleted (5 min, 10 000*g*, 25°C) and the EPS-containing supernatant was retrieved and pooled into new tubes. The EPS extract was stored at −20°C until further use.

#### 2.3.3. Activity assay

Soluble Pel substrate [α-1,4-poly(*N*-acetyl)galactosamine, p*K*_a_ ≃ 8], kindly provided by Novonesis and prepared as described above, was prepared at a concentration of 2 mg ml^−1^ in 20 m*M* sodium acetate buffer. Subsequently, 90 µl of the substrate solution was mixed with 10 µl enzyme solution at final concentrations of 0, 10 and 50 n*M* in 20 m*M* sodium acetate buffer. The mixtures were incubated at 30°C for 30 min with shaking at 750 rev min^−1^. Each enzyme concentration was tested in triplicate. MALDI-TOF analysis was performed using an AnchorChip target on a Bruker UltrafleXtreme MALDI-TOF/TOF instrument. Details of an additional assay, further confirming the Pel substrate composition of both α-1,4-linked galactosamine and *N*-acetyl­galactosamine, consistent with the analysis by Le Mauff *et al.*, 2022 ▸, are given in the supporting information (Le Mauff *et al.*, 2022 ▸).

#### 2.3.4. ITC binding assay

ITC was performed using a Malvern PEAQ ITC. The protein (*Tm*GH191 in size-exclusion buffer) was added to the cell at a final concentration of 50 µ*M* and GalNAC (Sigma–Aldrich) or GalN (Sigma–Aldrich) dissolved in the same buffer to a final concentration of 750 µ*M* was titrated into the protein. A total of 20 injections were carried out per run. A run with GAG preparations dissolved in buffer to a final concentration of 5 mg ml^−1^ was carried out in the same way.

### 2.4. Crystallization, data collection and structure determination

For all three enzymes, computations were carried out using programs from the *CCP*4 suite (Agirre *et al.*, 2023 ▸), via the *ccp*4*i*2 (Potterton *et al.*, 2018 ▸) or *ccp*4*i* (Potterton *et al.*, 2003 ▸) interfaces, unless stated otherwise. Data-collection and processing and final refinement statistics are given in Table 1 ▸.

#### 2.4.1. *Tm*GH191

Crystallization of *Tm*GH191 was carried out using several commercially available crystal screens in vapour-diffusion format using an Oryx4 crystallization robot (Douglas Instruments). Drops with a total volume of 400 nl were set up by mixing reservoir and protein solution in a 1:1 ratio using an MRC 2-well crystallization plate. The drops were equilibrated against 60 µl reservoir solution. Several hits were obtained in conditions with neutral to basic pH and various high-molecular-weight PEGs. Crystals were optimized in a 24-well hanging-drop format using Linbro plates. The crystals used for data collection were grown in 0.1 *M* HEPES pH 7.5, 14% PEG 8000.

Diffraction data were collected on beamline 14.1 at HZB Berlin and beamline P11 at DESY Hamburg at 100 K to a maximum resolution of 2.5 Å. The data were integrated using *XDS* (Kabsch, 2010 ▸) using *XDSGUI* (Brehm *et al.*, 2023 ▸), the the final space group was selected using *POINTLESS* and data were scaled with *AIMLESS* via the *ccp*4*i*2 interface (Potterton *et al.*, 2018 ▸; Evans & Murshudov, 2013 ▸). Statistics indicated that the crystals suffered from moderate twinning. The structure was solved by molecular replacement with *MOLREP* (Vagin & Teplyakov, 2010 ▸) using the model of *Tm*GH191 deposited in the PDB by the Joint Center for Structural Genomics (JCSG) with no related publication (PDB entry 2aam), which was converted to a polyalanine chain to reduce the potential model bias expected due to a sequence identity of 100%. *Coot* (Casañal *et al.*, 2020 ▸) and *REFMAC*5 (Murshudov *et al.*, 2011 ▸) were used for subsequent iterative model correction and refinement.

#### 2.4.2. Env-GH191

Initial crystallization of Env-GH191 was carried out in a number of commercial screens using sitting-drop vapour diffusion with drops set up using a Mosquito robot (SPT LabTech, United Kingdom) with 150 nl protein solution plus 150 nl reservoir solution in 96-well format plates (MRC 2-well crystallization microplates, Swissci, Switzerland) equilibrated against 54 µl reservoir solution. Some minor non-diffraction-quality hits were obtained in the ammonium sulfate screen (Qiagen): this was followed by extensive optimization. The final crystals were very thin inter-grown plates that were obtained using 1.6% ammonium sulfate, 3% PEG 1K plus 100 µl glycerol on top in a 24-well tray (Linbro) in a hanging-drop setup, with a crystallization drop consisting of 0.5 µl protein solution and 0.5 µl well solution, and 0.5 ml in the well. Diffraction data were collected on beamline I04-1 at the Diamond Light Source at 100 K, processed with *XDS* (Kabsch, 2010 ▸) as incorporated in *xia*2 (Gildea *et al.*, 2022 ▸) and scaled with *AIMLESS* (Evans & Murshudov, 2013 ▸).

#### 2.4.3. Env-GH191: structure solution and crystal pathology

The structure was solved by molecular replacement (MR) with *MOLREP* (Vagin & Teplyakov, 2010 ▸) using the structure of a putative glycosidase (Tm1410) from *T. maritima* at 2.20 Å resolution (PDB code 2aam) as the search model. The initial attempt at structure solution was made in space group *C*222_1_ and resulted in one positioned copy of the search model (solvent content 68%). While the resulting model consisted of disconnected molecular layers, a second copy could not be positioned because of packing constraints. With the data reprocessed in space group *P*2_1_, three copies of the monomer were positioned (solvent content 51%). In this solution, the symmetry-related molecules formed a three-dimensional net without significant clashes, as expected for a correct MR solution. However, the electron density for chain *C* was poor and the difference map was indicative of an alternative conformation of the entire chain. Refinement and model correction of the *P*2_1_ model was conducted in a conventional manner, with chain *A* being corrected and copied to *B* and *C* using *Coot* (Casañal *et al.*, 2020 ▸).

To visualize the organization of the crystal, a model was generated from the *C*222_1_ and *P*2_1_ models using *Coot*. The new model had space group *C*222_1_ and *a*′ = 125.52 Å, which is three times larger than the *a* length of 41.84 Å for the original *C*222_1_ model. To accommodate the two alternative conformations, four chains (*A*, *B*, *C* and *D*) were modelled with a solvent content of 57% in the asymmetric unit; this is referred to in the following as the triple-cell model. This triple-cell model was assembled by superposing the *P*2_1_ structure (containing chains *A*, *B* and *C*) onto the *C*222_1_ structure (containing chain *A* only) using chain *A*, deleting chain *B*, renaming chain *C* to *D*, and adding chains *B* and *C* by translating chain *A* by *a* and 2*a*.

**Figure 1:**
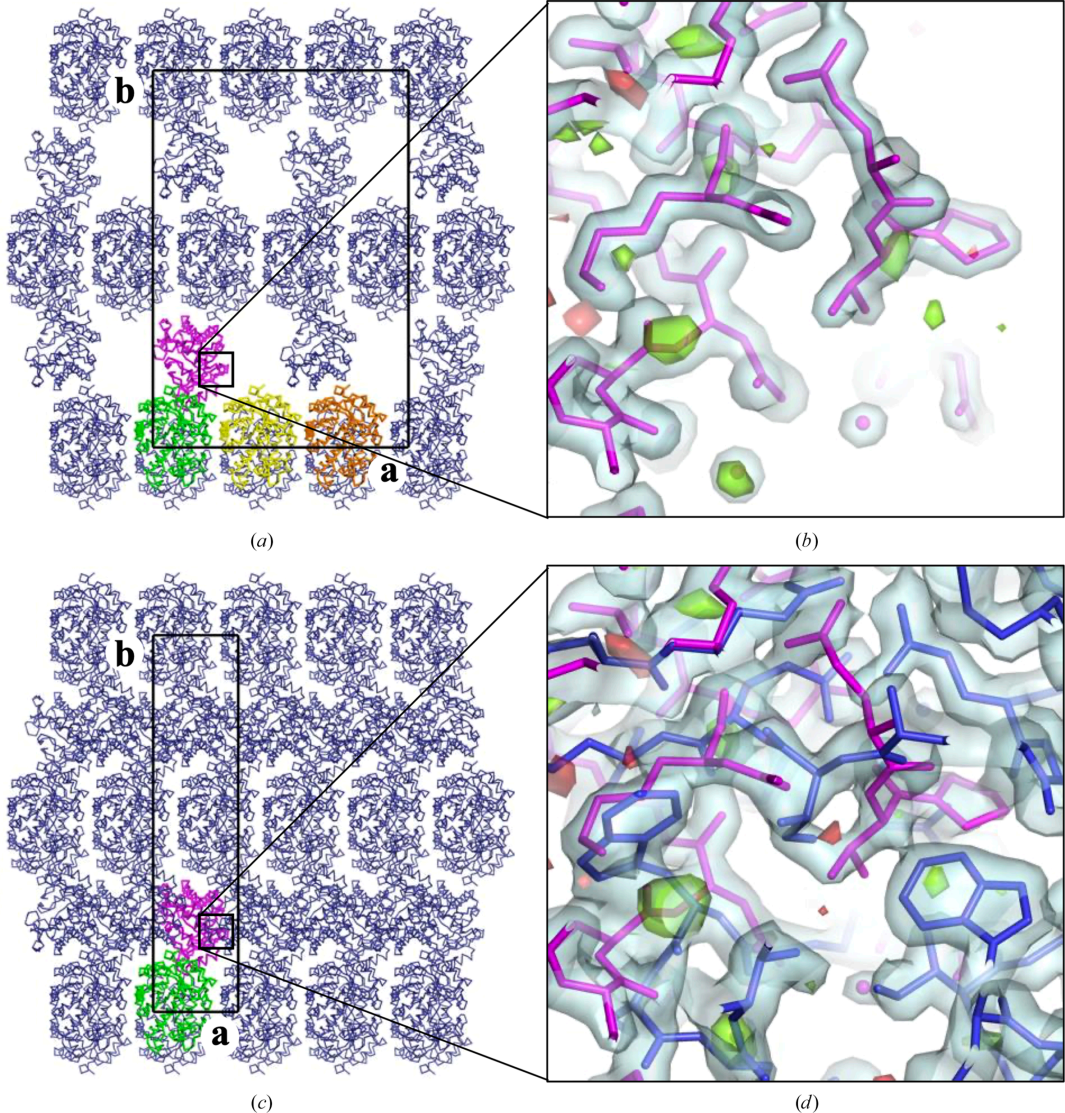
Crystal pathology. Models of the Env-GH191 crystal structure. (*a*, *b*) Triple-cell model representing an ordered fragment of the actual crystal structure and used for map deconvolution and model building; (*c*, *d*) a convoluted crystal model with unit-cell dimensions determined during data processing, where chain *B* has occupancy 1/3 and overlaps with one of its symmetry equivalents. The maps in (*b*) and (*d*) are for the areas indicated by the black boxes in (*a*) and (*c*). The contour level of the 2*F*_obs_ − *F*_calc_ map in (*d*) is 0.5σ, three times lower than in (*b*), to compensate for the partial occupancy of chain *B* in the convoluted crystal model. Chains forming the asymmetric unit in (*a*) and (*c*) are highlighted. Chains *A*, *B* and *C* shown in green, yellow and orange, respectively, in (*a*) and chain *A* (green) in (*c*) represent ordered layers of molecules, and chains *D* (magenta in both panels) represent the sparse layers. In the sparse layer of the real crystal, the voids and allowed orientations of molecules occur in random order. This figure was made using *PyMOL* (Schrödinger; DeLano, 2002 ▸).

Consider a row of molecules from the triple-cell model that includes chains *A*, *B* and *C* and their symmetry equivalents generated by crystallographic translations along *a*′ (Fig. 1 ▸*a*). There are two types of contact that chains from this row can make with chains from the adjacent space row: contact as shown between *A* and *D* and contact as shown between *C* and the symmetry equivalent of *D* generated by screw twofold rotation. In addition, there are chains that make no tight contacts with the sparse layer, as illustrated by chain *B*. The triple-cell model clearly exhibits all three possibilities, alternating along *a*. In a real crystal these three possibilities are likely to occur in random order.

Other crystal models can be generated that would have different regular arrangements of molecules in the sparse layer, but the triple-cell model was chosen because it has the smallest possible asymmetric unit. The ambiguity in the choice of a representative crystal model is eliminated if the triple-cell model is collapsed back to the original unit cell, as shown in Fig. 1 ▸(*c*), where chain *A* and its symmetry equivalents represent well ordered layers, while chain *B* overlaps with one of its symmetry equivalents and, together with all of its symmetry equivalents, represents the sparse layers.

The triple-cell model was originally constructed for presentation purpose only, but turned out to be a convenient tool for refinement and model correction as it allowed deconvolution of the electron density from molecules overlapping in the small cell and, as a result, ensured that conformational differences in chains belonging to both well ordered and sparse layers are accounted for. This part of the work has little relevance to the biological aspect of the article, but provides a paradigm for tackling the refinement of other partially disordered structures. The key features of the procedure are outlined below.

The data processed in *C*222_1_ were re-indexed (using the *REINDEX* program from the *CCP*4 suite) to give *C*222_1_ data with *a* = 125.52 Å and completeness 1/3. Using these data, the triple-cell structure was iteratively corrected using *Coot* (Casañal *et al.*, 2020 ▸) and refined using *REFMAC*5 (Murshudov *et al.*, 2011 ▸). In each round of model correction, adjustments were made in chains *A* and *D*, and *A* was copied to *B* and *C*. Such model building and refinement effectively deconvolute the electron-density maps from overlapping molecules because any atom can be modelled in three symmetry-independent positions to give the same contribution to reflections 3*h*, *k*, *l* for which there are observations. This implicit deconvolution of the electron density worked very well for the Env-GH191 structure and made it possible to comfortably correct all four chains including the one from a disordered layer, as well as to model water and sulfate molecules. The procedure has several features worth highlighting. The free-*R* flag was generated for all reflections, measured or not. This invokes a *REFMAC*5 output mode, used for map calculation, where *F*_calc_ values are substituted for missing *F*_obs_ values. Here we skip the fine details of likelihood-based map calculations to conclude that, in the first approximation, for missing observations *F*_calc_ are used instead of 2*F*_obs_ − *F*_calc_in the calculation of 2*F*_obs_ − *F*_calc_-type maps and, at the same time, the missing observations do not contribute to *F*_obs_ − *F*_calc_-type maps because *F*_obs_ − *F*_calc_ is zero. As a result, with 2/3 of the observations missing, the 2*F*_obs_ − *F*_calc_ map matches the model in correctly modelled places unusually well (Fig. 1 ▸*b*). However, the model bias is not as dramatic as one might expect. Both *F*_obs_ − *F*_calc_ and 2*F*_obs_ − *F*_calc_ maps for the unmodelled parts were good enough for manual building (although much less clear than in conventional refinement against more complete data) and the wrongly modelled residues were easy to recognize. For comparison, refinement using the original *C*222_1_ small-cell data and model building using overlapping maps was also tried, but rebuilding with deconvoluted maps turned out to be considerably more straightforward and reliable. However, in both cases the extensive use of validation tools in *Coot* was critical for avoiding overlooked model errors and NCS restraints were used during refinement. The final triple-cell model was collapsed into a smaller unit cell with chains *A* and *B* assigned with occupancies of 1 and 1/3, respectively (Figs. 1 ▸*c* and 1 ▸*d*). One run of *REFMAC*5 was carried out with the collapsed model before deposition in the PDB. The PDB file with the final triple-cell model and the corresponding MTZ file with map coefficients are available as supporting information for this article.

#### 2.4.4. *Fs*GH114

Initial crystallization of *Fs*GH114 was carried out as for Env-GH191. The first hits were obtained in PACT screen conditions D5, D6 and E5 (0.1 *M* MMT buffer pH 8 or 9, 20% PEG 1500 and 0.2 *M* sodium nitrate, 20% PEG 3350, where MMT buffer is produced by mixing dl-malic acid, MES and Tris base in the molar ratio 1:2:2 dl-malic acid:MES:Tris base). These initial crystals were used to prepare seeding stock, and microseed matrix screening (MMS; D’Arcy *et al.*, 2014 ▸) was carried out using an Oryx robot (Douglas Instruments) according to published protocols (Shah *et al.*, 2005 ▸; Shaw Stewart *et al.*, 2011 ▸). Briefly, crystals were transferred onto a glass slide, crushed and collected in a Seed Bead (Hampton Research) with 50 µl well solution added, vortexed for 1 min and used as an initial seeding stock: unused seeding stocks were stored at −20°C for later experiments. Following MMS, the final crystals were obtained in Index condition A6 (0.1 *M* Tris pH 8.5, 2 *M* ammonium sulfate), with 1 m*M* TEW [Anderson–Evans polyoxotungstate 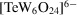 (TEW), Jena Bioscience] added to the protein solution prior to crystallization.

Data were collected on beamline I04 at the Diamond Light Source at 100 K, processed by *DIALS* (Winter *et al.*, 2018 ▸) within the *xia*2 pipeline (Gildea *et al.*, 2022 ▸; Winter *et al.*, 2013 ▸) and scaled with *AIMLESS* (Evans & Murshudov, 2013 ▸). The structure was solved by *MOLREP* (Vagin & Teplyakov, 2010 ▸) using PDB entry 6oj1 as a model. *Coot* (Casañal *et al.*, 2020 ▸) and *REFMAC*5 (Murshudov *et al.*, 2011 ▸) were used for subsequent iterative model correction and refinement.

### 2.5. Docking

*AutoDock Vina* (Trott & Olson, 2010 ▸; Eberhardt *et al.*, 2021 ▸) was used to create models of the complexes between a galactosaminogalactan and Env-GH191 and *Fs*GH114, respectively. As mentioned above, galactosaminogalactans (GAGs) are positively charged extracellular polysaccharides composed of varying amounts of galactose (Gal), *N*-acetyl­galactosamine (GalNAc) and galactosamine (GalN). The N-unsubstituted galactosamines are probably randomly distributed (Takada *et al.*, 1981 ▸), but polygalactosamine and an alternating structure of GalNAc and GalN were chosen as model substrates for the docking, as supported by Le Mauff *et al.* (2022 ▸), who identified the Pel substrate as a GalN–GalNAc dimer repeat (Le Mauff *et al.*, 2022 ▸). *PolysGlycanBuilder* (https://glycan-builder.cermav.cnrs.fr; see Lal *et al.*, 2020 ▸) was used to create heptasaccharides with the structure α-1,4-poly-GalN and poly(α-GalN-1,4-GalNAc) (alternating): Dgal*p*N-α1-4-Dgal*p*NAc-α1-4-Dgal*p*N-α1-4-Dgal*p*NAc-α1-4-Dgal*p*N-α1-4-Dgal*p*NAc-OH.

The *A* chains of the structures of Env-GH191 and *Fs*GH114 were stripped of all nonprotein atoms and supplied to *AutoDock Vina*, along with the heptasaccharide model. All single bonds in the oligosaccharides were allowed to rotate during the docking simulations, whereas the side chains were fixed. 1000 poses were created for each complex.

## 3. Results and discussion

### 3.1. Phylogenetic analysis, cloning and expression of putative endo-galactosaminidases and formation of a new GH family

**Figure 2:**
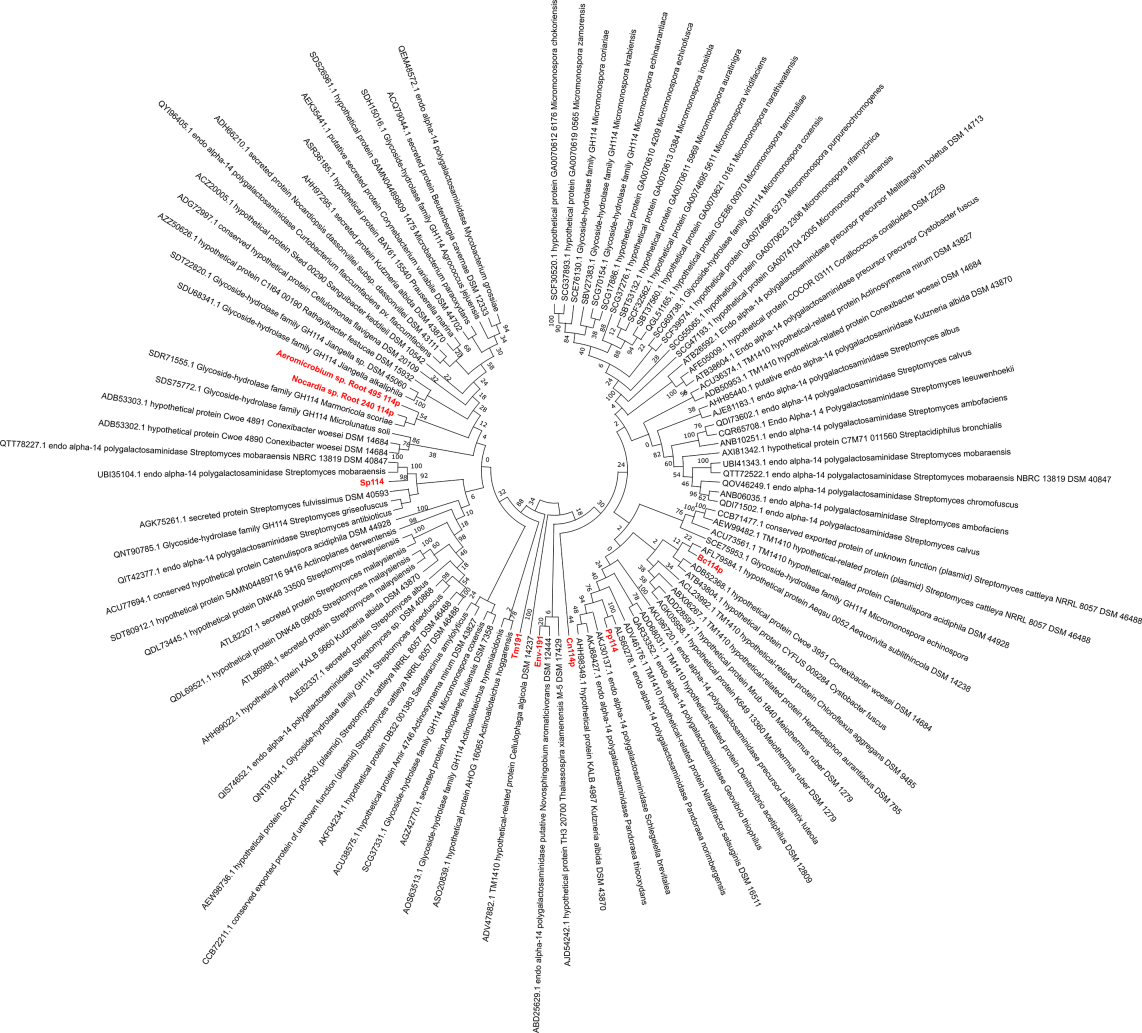
Phylogenetic tree of bacterial putative GH114 producers. Phylogenetic tree of sequences, extracted from the CAZy database (Drula *et al.*, 2022 ▸), of bacteria for which the strain is available from the DSMZ. The alignment and tree were generated with *MEGA* (Tamura *et al.*, 2021 ▸).

We analysed sequences from bacteria available in the repository of the German Collection of Microorganism and Cell Cultures GmbH (DSMZ) to identify potential promising galactosaminidases (Fig. 2 ▸), in particular from family GH114, due to the expected selectivity for deacetylated stretches of the mature exopolysaccharide. Furthermore, we included the *P. putida* variant that has been functionally characterized (Tamura *et al.*, 1988 ▸) and an enzyme from *T. maritima* which showed similarity to PelA on a structural level (Bamford *et al.*, 2015 ▸), as well as a sequence isolated from an environmental sample in Denmark and attributed to be a galactosaminidase with a Pfam domain (PF03537) that shows similarity to the GH114 family hydrolase domain. Additionally, a fungal enzyme from *F. solani* with 33% sequence identity to Ega3 was included. The sequence analysis showed that we cover a wide sequence range (Fig. 2 ▸). Surprisingly, two members from our selection, the environmental sample enzyme and the *T. maritima* enzyme, had not been assigned into CAZy GH114. Thus, based on the sequences and subsequently shown activity (see below) for one family member, we propose the formation of a new CAZy family GH191 distinct from the other known endo-galactosaminidase families GH114, GH135 and GH166.

To gain further insight into the endo-galactosaminidase families and their interrelationship, we selected potential candidate genes from *B. cepacia* DSM7288, *C. necator* H16 DSM428, *Nocardia* sp. Root240 DSM102477, *Aeromicrobium* sp. Root495 DSM102310, *S. platensis* DSM40041 and *F. solani* from the family GH114 endo-galactosaminidases and the environmental sample enzyme from Denmark, together with the *T. maritima* enzyme, as members of the new GH191 family for further characterization. All attempts to produce the enzymes selected from the GH114 family in soluble form in *E. coli* failed. A variant of *S. platensis* GH114 (*Sp*GH114) could be obtained in soluble form as an N-terminal MBP fusion. After cleavage of the fusion protein with TEV protease, we observed precipitation. The subsequent size exclusion showed only large aggregates, indicating incorrect folding of *Sp*GH114. Independently, the two bacterial enzymes representing the new GH191 family, *Tm*GH191 and Env-GH191 from the environmental sample collected in Denmark, were produced recombinantly in *E. coli* and *B. subtilis*, respectively, in soluble form. The fungal GH114 enzyme from the filamentous fungus *F. solani*, which shows a sequence identity of 33% to Ega3 from *A. fumigatus*, could also be produced in soluble form. Thus, while we could produce two members of the new GH191 family in soluble form, and another fungal member of the GH114 family, bacterial endo-galactosaminidases of the GH114 family remain elusive.

### 3.2. Functional characterization

**Figure 3:**
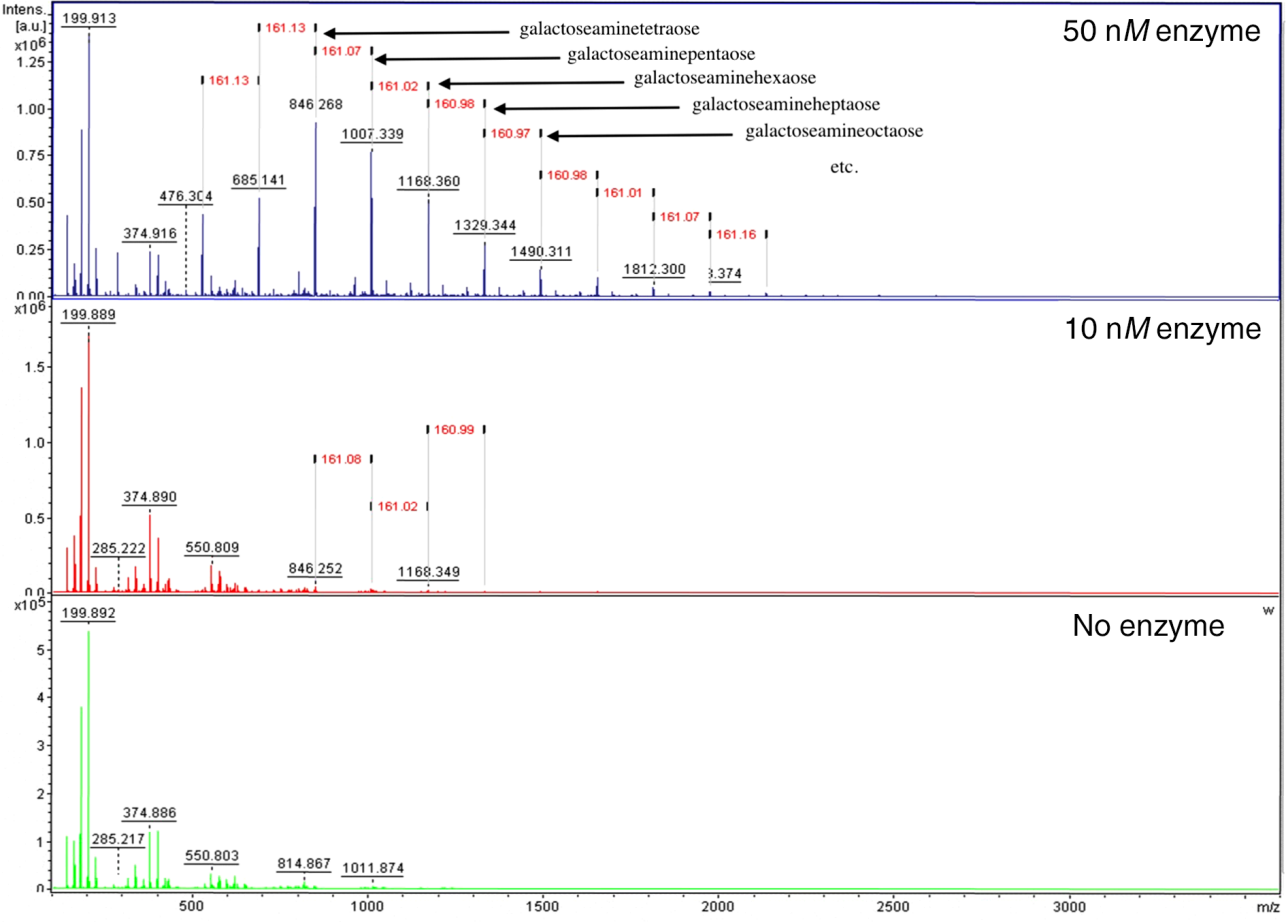
Activity measurements. The Pel substrate was mixed with different concentrations of the enzyme. The mixtures were incubated at 30°C for 30 min with shaking at 750 rev min^−1^. The bottom (green) panel corresponds to substrate with no enzyme added, the red panel has 10 n*M* enzyme added and the blue panel has 50 n*M* enzyme added. The figure demonstrates the activity of Env-GH191 towards α-1,4-poly(*N*-acetyl)galactosamine. The peaks correspond to different oligomerization states of the Pel substrate. Peaks with different degrees of polymerization (DP) are indicated, with DP4 corresponding to galactoseaminetetraose (sodiated; therefore Na^+^ is shown, but only for the first peak), DP5 to galactoseaminepentaose and so on. The MS fingerprint shows weight losses of ∼160 Da, corresponding to a galactosamine monomer.

To gain insight into the catalytic function of the two soluble enzymes of the new CAZy family GH191, we attempted to measure the activity against native GAG oligomers purified from the supernatant of *A. fumigatus* cell cultures as described previously (Lee *et al.*, 2016 ▸). All attempts for the *T. maritima* enzyme were unsuccessful. As a next step, we tried to measure binding with ITC using the purified GAG oligomers from *A. fumigatus* CEA10, galactosamine and *N*-acetyl­galactos­amine, both from Sigma–Aldrich. We could not detect any binding, or enzymatic cleavage of the GAG oligomers, for the *T. maritima* enzyme, which raises the question as to whether the *T. maritima* enzyme is an endo-galactosaminidase or whether GAG is not a substrate for this family. The enzyme from the environmental sample was tested with purified Pel and showed good activity towards the isolated Pel, with a preference for GalN-rich oligosaccharides (Fig. 3 ▸), confirming that the new family GH191 has members with galactosaminidase activity, with a preference for partially deacetylated stretches of Pel. It remains to be seen whether all members share activity on Pel substrate and how exactly the substrate-substitution pattern influences the activity. Furthermore, additional tests with native and synthetic substrates and substrate analogues will be required to determine whether the new family is also able to act on fungal GAGs or other similar structures. Cross-kingdom activity was previously observed for the GH166 PelA_H_ (Snarr *et al.*, 2017 ▸). For *Fs*GH114 no activity was seen using the isolated Pel substrate and further studies are necessary to establish the exact substrate specificity of *Fs*GH114.

### 3.3. Structure solution

Structure solution of the fungal *Fs*GH114 was straightforward by MR to a maximum resolution of 1.6 Å using Ega3 (PDB entry 6oj1) as a search model. However, the difference map indicates the presence of a fourth molecule in the asymmetric unit. If this molecule is modelled in the structure, it will overlap with its copy related by crystallographic twofold rotation. The extended structure can be refined if the fourth copy is assigned an occupancy of 0.5. Such refinement would have resulted in high *R* factors and poor density for the fourth copy. Therefore, the model with three copies was deposited and a relevant note was added to the PDB entry remarks.

The structures of both bacterial counterparts also had pathologies. The *Tm*GH191 crystals all showed a varying degree of twinning. The crystal organization is rather unusual. The structure has symmetry *P*2_1_ and pseudo-symmetry *P*6_3_, with crystallographic 2_1_ and pseudo-6_3_ axes nearly parallel and with a 1.7 Å C^α^ r.m.s.d. of the structure from its symmetrized *P*6_3_ version. The structure is composed of tightly packed trimers stacked on each other along their common pseudo-threefold axis. A pair of adjacent trimers form the asymmetric unit, with the two trimers having slightly different orientations (11.5° rotation around the common axis). The stacks of trimers pack into a sparse honeycomb-like structure with wide wells around the pseudo-6_3_ axis and a solvent content of 82.5%. Pseudosymmetry is not surprising with such a flexible frame. A side effect of symmetry reduction from *P*6_3_ to *P*2_1_ might have been twinning with three twin individuals related by a threefold rotation. The results of the *L*-test (Padilla & Yeates, 2003 ▸) vary over a wide range depending on the data-integration pipeline, all suggesting different degrees of twinning. However, these are most likely to be false positives caused by partial overlaps of neighbouring spots, as is typical for crystals with large cells, because no other significant evidence of twinning has been found: a small deviation from the nontwin reference in the *R*_merge_ (*POINTLESS*; Evans, 2011 ▸) and *H*-tests for threefold operations (*CTRUNCATE*; Winn *et al.*, 2011 ▸) can be attributed to pseudosymmetry. Moreover, the self-rotation function clearly shows no sixfold symmetry in the data: there are two close strong peaks, rather than six or one, in the χ = 60° section (*MOLREP*; Vagin & Teplyakov, 2010 ▸). Twin refinement introduced further controversy, as it reduced the *R* factors by approximately 0.05. However, Murshudov (2011 ▸) noted that when model errors reach a certain level, twin refinement can reduce the *R* factors regardless of whether the crystal is truly twinned (Murshudov, 2011 ▸). A theoretical boundary value of *R* ≃ 0.42 was derived for hemihedral twinning without pseudosymmetry. The authors suggest that this boundary may shift to significantly lower *R* factors in the presence of pronounced threefold pseudosymmetry. Consequently, twin refinement was not applied to the structures deposited in the PDB. Nonetheless, the authors recognize that the optimal approach may be more nuanced than a simple binary choice.

The crystal pathology of Env-GH191 was dealt with using a nonconventional procedure described in Section 2. This approach, in which an artificial larger cell is created to simplify refinement and model correction of the otherwise clashing whole-chain alternative conformations, should prove to be useful in solving similar partially disordered structures.

### 3.4. Structural analysis

**Figure 4:**
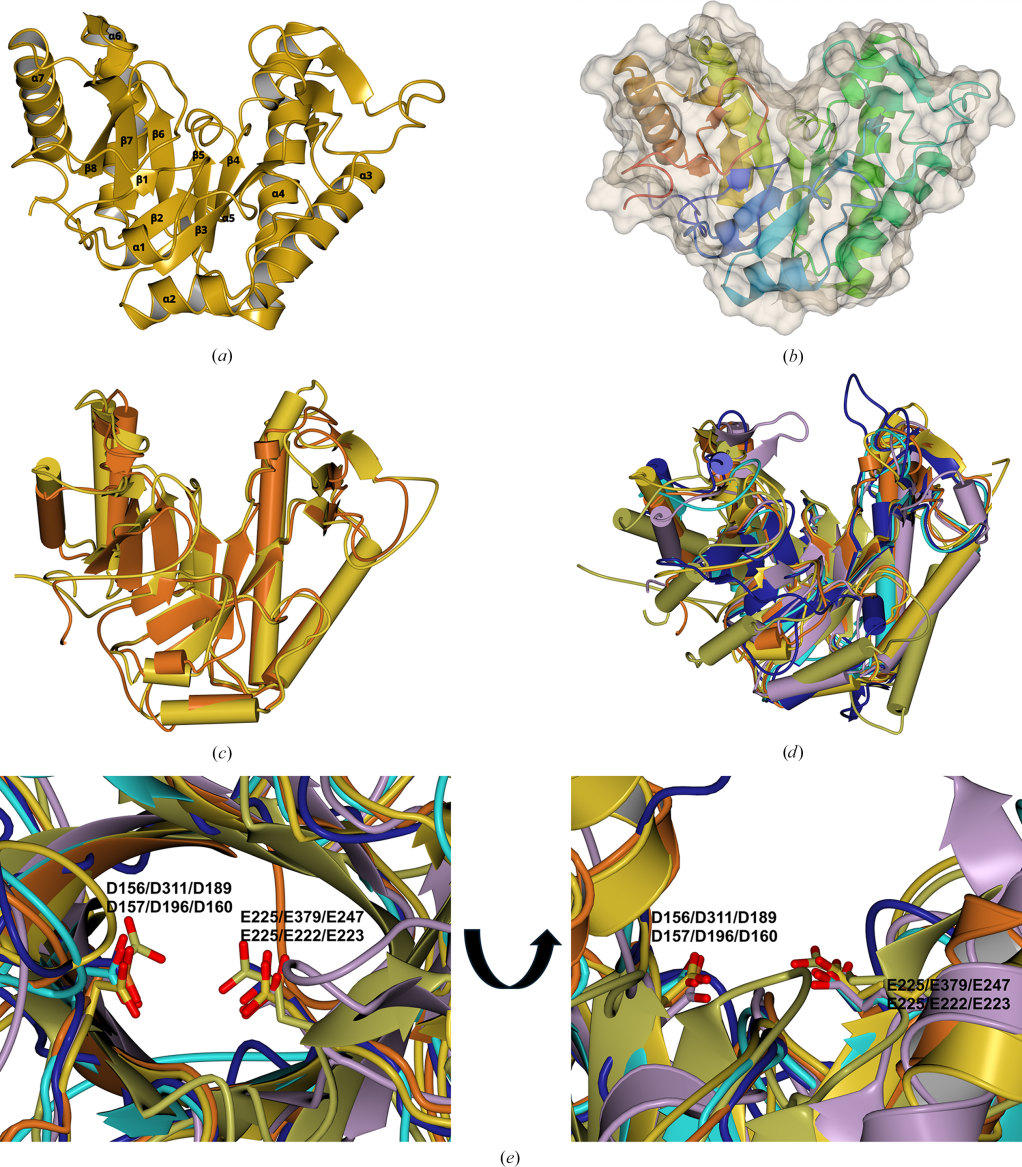
Structure of a bacterial GH191 enzyme. (*a*) Ribbon representation of *Tm*GH191 in light brown. (*b*) Ribbon representation of *Tm*GH191 coloured from the N-terminus to the C-terminus in blue to red, with the van der Waals surface in grey showing the substrate crevice in the middle of the protein on the C-terminal side of the barrel. (*c*) Overlay of *Tm*GH191 in light brown with Env-GH191 in dark brown. (*d*) Overlay of *Tm*GH191 in light brown with Env-GH191 in dark brown, *Fs*GH114 in dark blue, *Af*GH114 (Ega3; PDB entry 6oj1) in light blue, *Ac*Sph3 (PDB entry 5c5g) in gold and *Pa*PelA_H_ (PDB entry 5tcb) in lilac. (*e*) Close-up of the two active-site residues in the GH191 family and GH114 enzymes with the aspartate as the nucleophile and the glutamate as a general acid/base. The residue numbering is for *Tm*GH191, Env-GH191, *Af*GH114, *Fs*GH114, *Ac*Sph3 and *Pa*PelA_H_, respectively. This figure, as well as Figs. 5 ▸, 6 ▸ and 8 ▸, was made using *CCP*4*MG* (McNicholas *et al.*, 2011 ▸).

The GH191 enzymes share a common modified (β/α)_8_-barrel fold as the defining structural element for this family (Fig. 4 ▸). The two GH191 enzymes overlap well on each other, with an r.m.s.d. of 1.48 Å over 252 residues, while only having a sequence identity of 28%. Similarly, within the GH114 family, *F. solani* GH114 overlaps with Ega3 (*Af*GH114) with an r.m.s.d. of 1.49 Å over 218 residues, with a sequence identity of 35%. It is evident that families GH191 and GH114 share the same fold. Indeed, despite low sequence identities (∼13–27%) and higher r.m.s.d.s, with the largest difference of 3.01 Å for Env-GH191 and Ega, the overall fold is conserved (Table 2 ▸, Fig. 4 ▸*c*). The structural resemblance to the common (β/α)_8_-barrel holds true if one compares all families that have endo-galactosaminidase activity on extracellular cationic galactosamine-containing polysaccharides. Env-GH191, *Tm*GH191, Ega3, *Fs*GH114, Sph3 (GH135) and PelA_H_ (GH166) overlap with each other within a maximum r.m.s.d. of 3.09 Å (Figs. 4 ▸*d* and 5 ▸).

Whereas helices α1, α6 and α8 are absent in Ega3 (Bamford *et al.*, 2019 ▸), both bacterial GH191s contain the α1 helix as a structural element. *Fs*GH114 also lacks the first and sixth α-helices but has a C-terminal extension folding into two strands forming a short antiparallel β-sheet. All enzymes have a three-stranded β-sheet insertion between β3 and α3 which is thought to be important for ligand binding. In particular, a loop between β_i_2 and β_i_3 is observed in two conformations:‘open’ and ‘closed’. Both *Fs*GH114 and the fungal enzymes have a conserved disulfide pattern, whereas the GH191 enzymes show no conservation of the disulfide bonds, which are absent in the case of *Tm*GH191.

**Figure 6:**
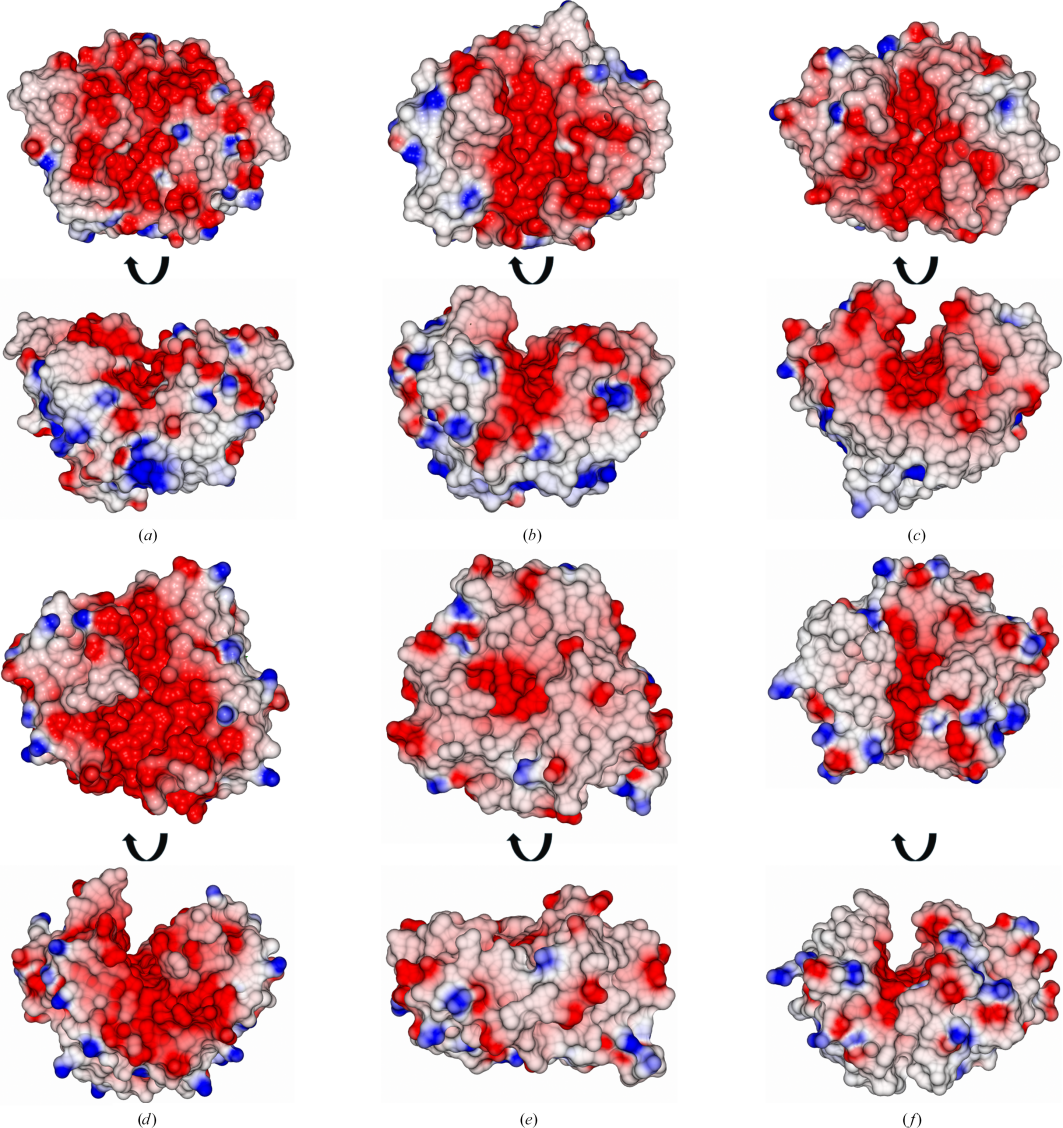
Substrate-binding sites of endo-galactosaminidase enzymes. (*a*) Substrate-binding site of *Tm*GH191. (*b*) Substrate-binding site of Env-GH191. (*c*) Substrate-binding site of Ega3. (*d*) Substrate-binding site of *Fs*GH114. (*e*) Substrate-binding site of Sph3. (*f*) Substrate-binding site of PelA_H_.

The putative active-site residues (Asp156/Glu225 for *Tm*GH191, Asp323/Glu391 for Env-GH191 and Asp157/Glu225 for *Fs*GH114) overlap well with the experimentally verified nucleophile and general acid/base Asp189/Glu247 in Ega3, Asp196/Glu222 in Sph3 and Asp160/Glu223 in PelA_H_. They are located on the C-terminal end of the barrel domain (Fig. 4 ▸*e*). Five of the six structures share the same negatively charged surface crevice assumed to be the substrate-binding site (Figs. 6 ▸*a*–6 ▸*f*). The negative charge is ideal for anchoring the positively charged substrate to the enzyme via charge interactions. Sph3 has a rather less charged surface due to its activity on neutral GlcNAcylated GAG (Bamford *et al.*, 2019 ▸). A closer inspection shows that the substrate crevice is partially blocked in ‘apo’ *Tm*GH191, especially over the active site via the loop between β_i_2 and β_i_3, with Trp121 bridging the active site and blocking access towards the putative −1 subsite (Figs. 6 ▸*a* and 7 ▸). This might explain the lack of activity of *Tm*GH191.

**Figure 7:**
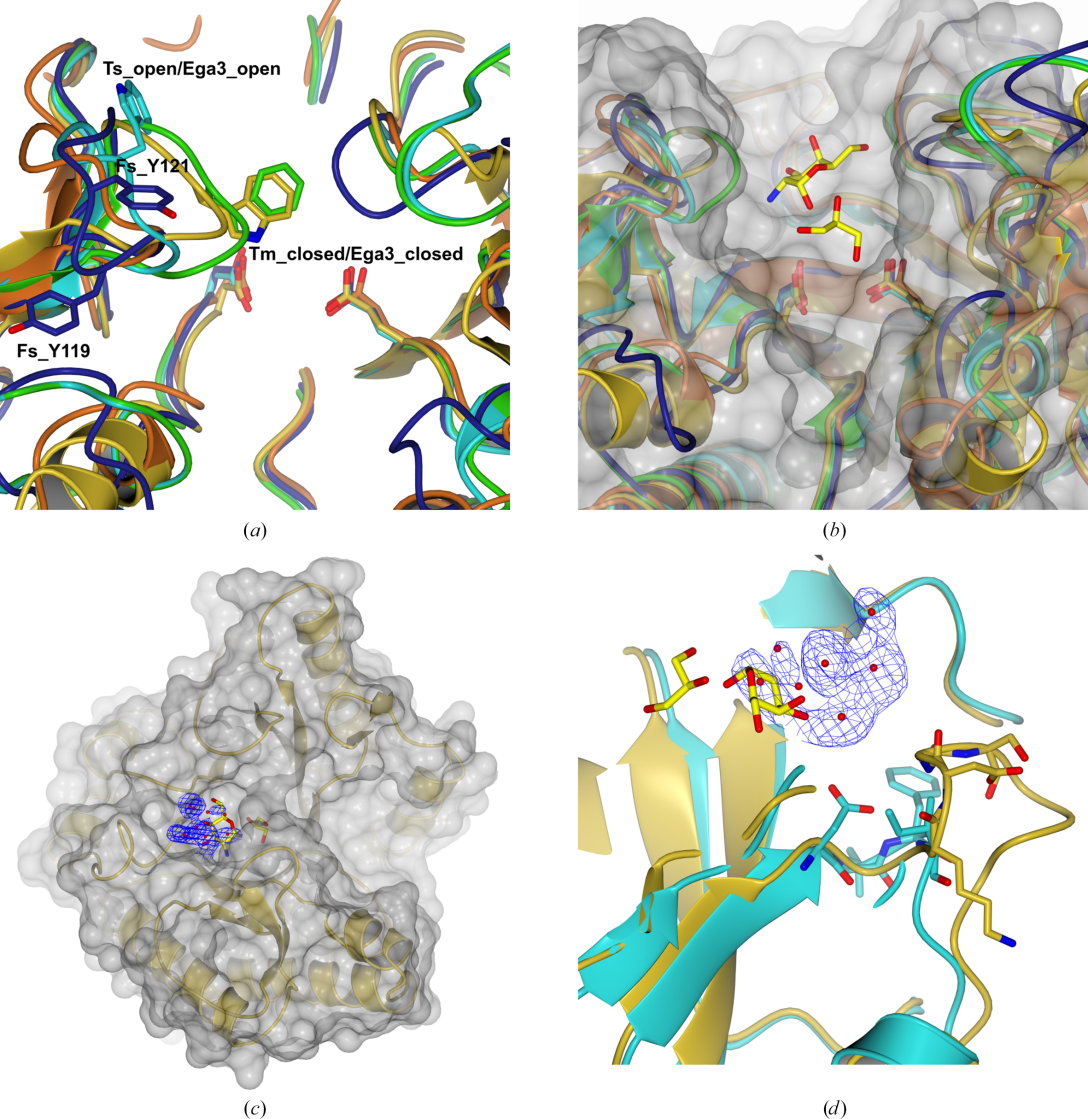
Substrate binding in family GH191 and GH114 enzymes. (*a*) Observed loop conformation of the 120-loop (*Tm*GH191 notation) in the different homologues. An open and closed conformation is observed. In *Fs*GH114 the prominent tip-residue tryptophan is replaced by two tyrosines. (*b*) Location of a glycerol in *Tm*GH191 (PDB entry 2aam) in subsite −1 and GalN in subsite −2 of *Af*GH114 (Ega3; PDB entry 6ojb). (*c*) Observed unexplained density in *Tm*GH191 located between subsite −2 and putative subsite −3. (*d*) Difference in conformation of the loop between *Tm*GH191 and *Af*GH114 (Ega3) around Asp64 (*Tm*GH191 notation) that partially closes the active crevice beyond subsite −3 in the bacterial members.

However, as mentioned above, it has been shown for Ega3 from the GH114 family that this loop, which is part of the β3 insertion, is a flexible element that opens and closes over the active site in response to substrate binding (Bamford *et al.*, 2019 ▸). In Ega3 (PDB entries 6oj1 and 6ojb), this loop, with Trp154 at its tip (corresponding to Trp121 and Trp288 in *Tm*GH191 and Env-GH191, respectively) moves 12.3 Å towards the ligand upon ligand binding (Fig. 7 ▸*a*). However, in *Tm*GH191 (PDB entry 2aam) no ligand was reported to have been added during crystallization. The *Tm*GH191 crystals prepared in this study were soaked with GalN and GalNAc, and a data set was obtained for a GalN-soaked crystal. While no distinct density for a bound carbohydrate was observed, difference density in the substrate-binding crevice of *Tm*GH191 suggests that something has bound, although it could not be conclusively modelled (Figs. 7 ▸*c* and 7 ▸*d*). It remains uncertain whether the loop only closes upon substrate binding or whether it adopts multiple conformational states in the absence or presence of the ligand. Some flexibility in the side chain of Trp121 was noted when comparing the native and soaked structures. However, it is unclear whether this reflects genuine increased flexibility or is merely a result of the higher overall *B*-factor distribution.

**Figure 8:**
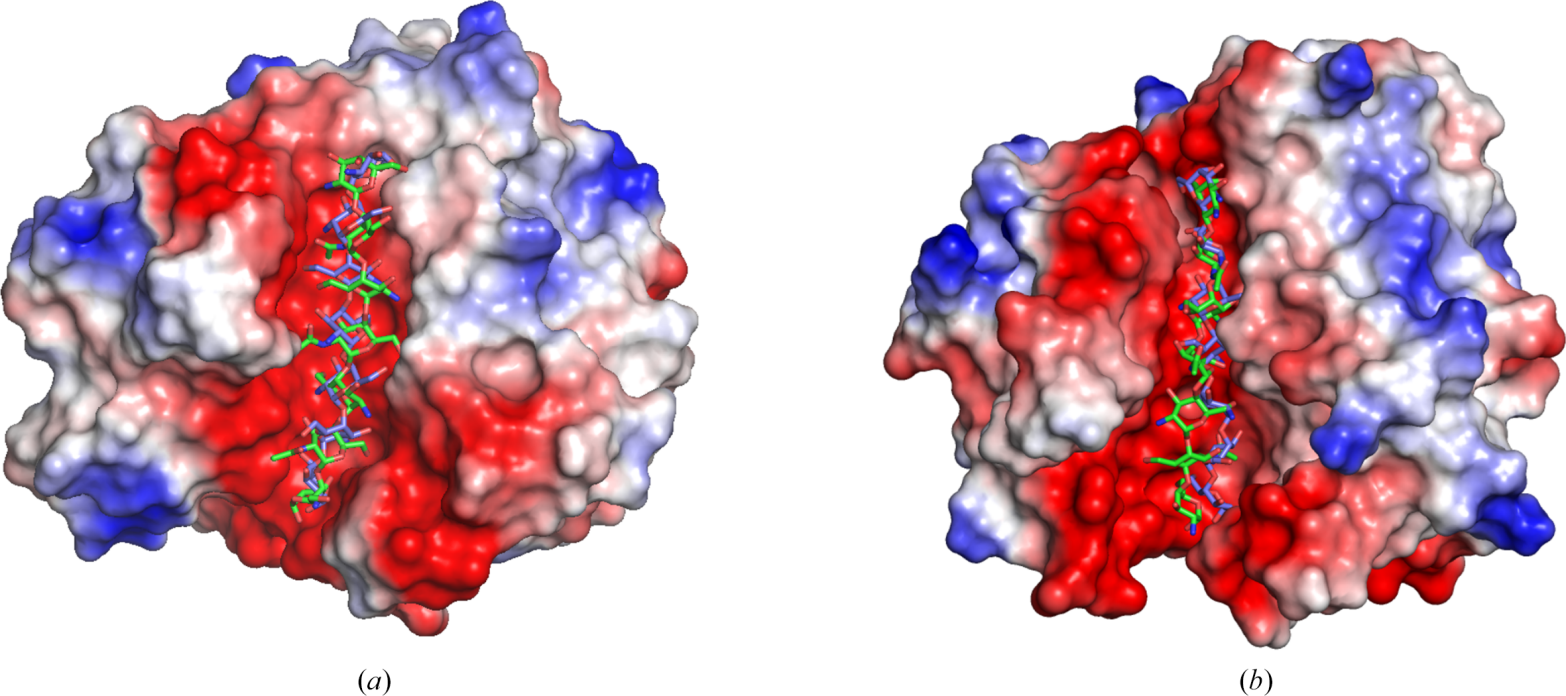
Substrate docking in family GH114 and GH191 enzymes. The nonreducing end is at the top and the reducing end is at the bottom of the crevice. (*a*) Best docking pose for GalNAc oligomers in green and GalN oligomers in blue in Env-GH191. (*b*) Best docking pose for GalNAc oligomers in green and GalN oligomers in blue in *Fs*GH114. This figure was made using *PyMOL* (DeLano, 2002 ▸).

Interestingly, the corresponding loop in *Fs*GH114 comprising residues 117–124 does not contain a conserved tryptophan but rather has two tyrosines (Tyr119/Tyr121) on its tip and is in an open conformation (Fig. 7 ▸*a*). One of these tyrosines or both could play the same role as the tryptophans in the other three enzymes (Tyr121 is poorly defined in the present structure). A flexible loop with aromatic tip residues (Tyr and Phe) over the active site is also found in the family of amylomaltases, which are active on the structurally related substrate amylose (Roth *et al.*, 2017 ▸). Furthermore, a loop closure is implicated to support the inherent transglycosylation reaction in glycoside hydrolases (van der Veen *et al.*, 2001 ▸; Llopiz *et al.*, 2023 ▸), a reaction that is also observed in GH114 enzymes (Tamura *et al.*, 1992 ▸) and may also be present in GH191, GH135 and GH166 enzymes, although this has not yet been experimentally proven. To date, only one structure of Ega3 (PDB entry 6ojb) has a carbohydrate monomer (GalN) in subsite −2. Efforts to obtain a complex of *Tm*GH191 soaked with GalN failed. However, difference electron density approximating to a putative −3 subsite is observed, which could not be assigned to any component of the crystallization cocktail. In the deposited structure of *Tm*GH191 (PDB entry 2aam) a glycerol moiety is bound in subsite −1 hydrogen-bonded to Asp155 and Glu223. Interestingly, the electron-density map of PDB entry 2aam revealed electron density similar to the difference density in our structure for the crystal soaked with GalN. In PDB entry 2aam this was modelled as an ‘unknown ligand’ represented by linked oxygens arranged in a resemblance to a sugar shape. It remains to be seen what compound may have bound in both cases. A comparison of the substrate-binding crevices shows that beyond subsite −3 in both bacterial structures a loop around Asp64 (*Tm*GH191 notation), connecting β2 to α2, partially closes the crevice. In both fungal GH114 structures the crevice is more open and extended, potentially allowing substrate binding beyond −3 and up to −5 (Figs. 7 ▸*c* and 7 ▸*d*). The acceptor side of the substrate crevice in *Fs*GH114 is flanked by two elongated loops comprising residues 164–168 between β4 and α4 and C-terminal to β6 residues 231–239. Thus, overall the crevice of *Fs*GH114 is deeper and more pronounced compared with the bacterial GH191 enzymes, but also with Ega3 (Fig. 6 ▸). To gain better insight into the substrate interactions, we docked the substrate into Env-GH191 and *Fs*GH114. In both cases up to seven subsites could be identified. Whereas the Env-GH191 binding crevice seem to better fit the galactosamine oligomer, the *Fs*GH114 crevice can accommodate either an acetylated or a non-acetylated GAG oligomer equally well (Fig. 8 ▸). In line with our observation that the loop around Asp64 (*Tm*GH191 notation) may preclude binding beyond subsite −3 for the bacterial enzymes, the cleavage will occur closer to the non­reducing end of the bound substrate. Our determined structures of the GH191 members and characterized GH166 members share the same β3 insertion, including the flexible loop over the active site. Similarly, the loop around Asp64 (*Tm*GH191) forming a barrier towards the nonreducing end of the substrate crevice is found in GH166 PelA_H_ and not in the characterized GH135 Sph3. Thus, the newly characterized GH191 enzymes seem more similar to PelA_H_, the founding member of the GH166 family. This is also reflected in the specificity towards the substrate. While the GH135 and GH166 families are both retaining α-*N*-acetyl­galactos­aminidases, GH166 enzymes also have a preference for partially deacetylated stretches compared with the GH135 enzymes (Le Mauff *et al.*, 2019 ▸), which is in line with the observed specificity of Env-GH191.

## 4. Conclusions

Our knowledge of endo-galactosaminidases involved in the degradation of extracellular fungal GAG and bacterial Pel is still very limited. So far, for each current family within the CAZy database two or fewer members have been characterized, with only one member each being structurally characterized in more detail. We aimed to further investigate these underexplored families by characterizing a number of potential candidates. While initially we wanted to focus on GH114 enzymes, we uncovered a new CAZy family, GH191, with endo-galactosaminidase activity. Activity measurements for one member, Env-GH191, indicate a preference for bacterial Pel polysaccharide. A detailed analysis will be necessary to fully characterize the substrate scope of this family in the future. Structures were solved for three proteins: two members of the new family and a new fungal member of family GH114. The TIM-barrel core structure with fully conserved active-site residues and a negatively charged substrate crevice is conserved among all the families. A comparison with other GAG and/or degrading glycoside hydrolase families revealed that the new enzymes all have a β3 insertion subdomain containing a flexible loop similar to the GH114 and GH166 enzymes. Furthermore, the bacterial members have a loop preventing the extension of the substrate crevice at the nonreducing end, while the fungal *Fs*GH114 enzyme has a less restricted substrate crevice more similar to the GH135 *N*-acetylgalactosaminidases. However, the new enzyme family shows a higher overall similarity to the fungal GH114 and the bacterial Pel-degrading family GH166, which also act on GAGs with a higher deacetylation grade. It remains to be seen whether more family members of this new family can be produced in sufficient amounts for detailed functional and structural characterization to shed further light on the still elusive substrate-binding mode and catalytic mechanism of this new family of enzymes. This might also allow us to better understand the catalytic mechanism of families GH114, GH135 and GH166, for which the substrate-binding mode is also elusive. In addition, the definition of the catalytic mechanism will help to clarify the interrelationship of families GH114, GH135, GH166 and GH191 as a potential new clan of GAG- and Pel-degrading enzymes, as already suggested previously for GH114 and GH166 (Le Mauff *et al.*, 2019 ▸). Moreover, the studies might help to unravel the role of and the reason for the occurrence of four families of endo-α-1,4-galactosaminidases in the bacterial kingdom. Finally, the approach to refinement and model building for a structure with partial disorder that was successfully implemented in this work and outlined in Section 2 ▸ is of separate value and could be recommended for use in similar cases of crystal pathology.

## Supplementary Material

PDB reference: GH191 enzyme from *Thermotoga maritima*, 9eux

PDB reference: soaked with galactosamine, 9euz

PDB reference: GH114 enzyme from environmental sample, 9ep5

PDB reference: GH191 enzyme from *Fusarium solani*, 9ep6

Supplementary Methods and Supplementary Figure S1. DOI: 10.1107/S2059798325002864/rr5250sup1.pdf

PDB and MTZ files for the final triple-cell model. DOI: 10.1107/S2059798325002864/rr5250sup2.zip

X-ray diffraction images for GH114 enzyme from environmental sample.: https://doi.org/10.5281/zenodo.10936959

X-ray diffraction images for GH191 enzyme from Fusarium solani.: https://doi.org/10.5281/zenodo.11011296

X-ray diffraction images for GH191 enzyme from Thermotoga maritima.: https://doi.org/10.5281/zenodo.14389479

## Figures and Tables

**Figure 5:**
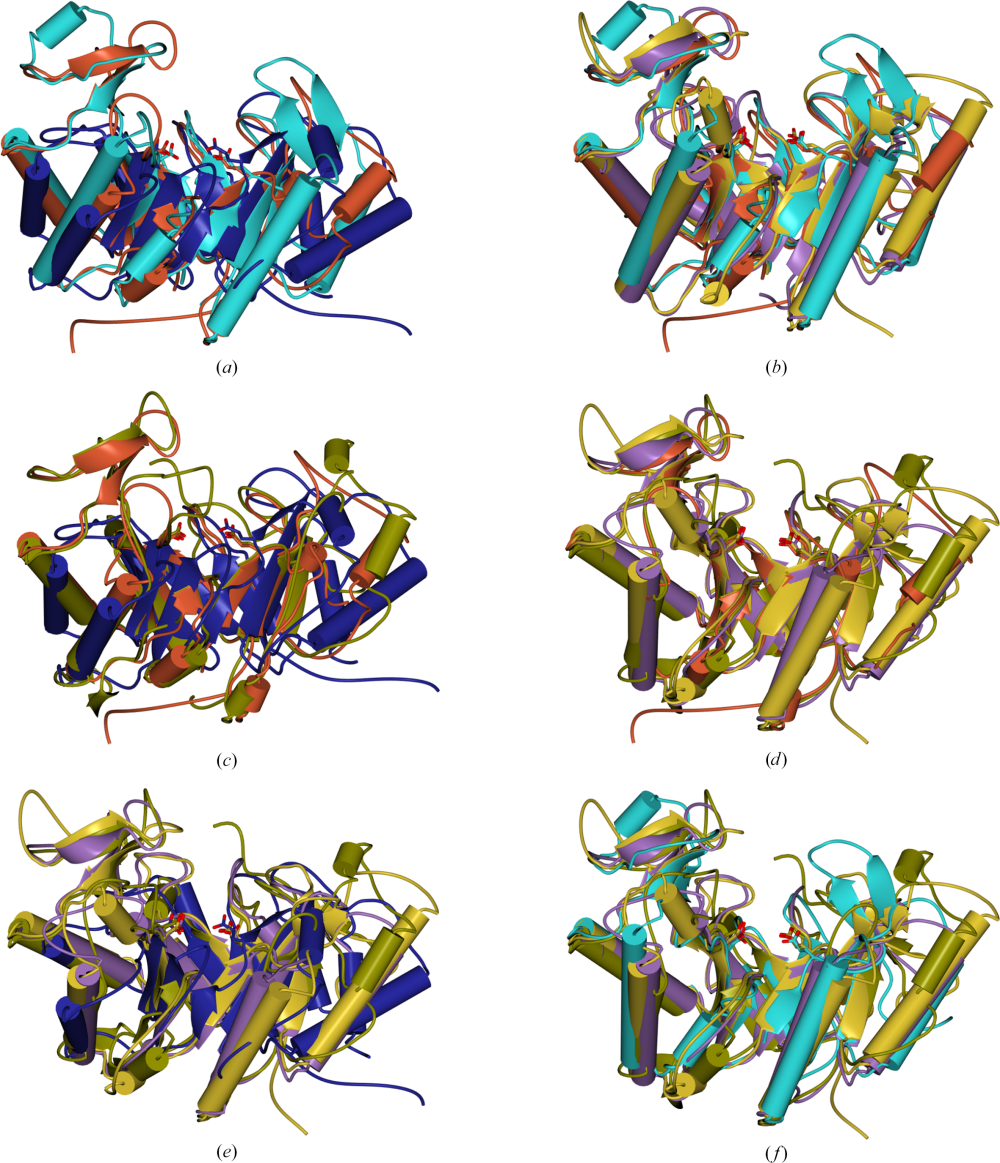
Structural comparison of galactosaminogalactan-degrading glycoside hydrolase families. (*a*) Overlay of GH114 Ega3 from *A. fumigatus* in red brown, GH135 Sph3 from *A. clavatus* (PDB entry 5c5g) in dark blue and GH166 PelA_H_ from *P. aeruginosa* (PDB entry 5tcb) in light blue. The catalytic residues are shown in stick representation. (*b*) Overlay of all bacterial enzymes. The *T. maritima* enzyme is in wheat, the Env enzyme is in purple and GH166 PelA_H_ from *P. aeruginosa* (PDB entry 5tcb) is in light blue. The catalytic residues are shown in stick representation. (*c*) Overlay of fungal enzymes: GH114 Ega3 from *A. fumigatus* in red brown, GH135 Sph3 from *A. clavatus* (PDB entry 5c5g) in dark blue and the *F. solani* enzyme in moss green. The catalytic residues are shown in stick representation. (*d*) Overlay of all enzymes characterized in this work with Ega3. The *T. maritima* enzyme is in wheat, the Env enzyme is in purple, the *F. solani* enzyme is in moss green and GH114 Ega3 from *A. fumigatus* is in red brown. The catalytic residues are shown in stick representation. (*e*) Overlay of all of the structurally characterized enzymes with GH135 family Sph3. The *T. maritima* enzyme is in wheat, the Env-GH191 enzyme is in purple, the *F. solani* enzyme is in moss green and GH135 Sph3 from *A. clavatus* (PDB entry 5c5g) is in dark blue. The catalytic residues are shown in stick representation. (*f*) Overlay of all of the structurally characterized enzymes with GH135 family Sph3. The *T. maritima* enzyme is in wheat, the Env enzyme is in purple, the *F. solani* enzyme is in moss green and GH166 PelA_H_ from *P. aeruginosa* (PDB entry 5tcb) is in light blue. The catalytic residues are shown in stick representation.

**Table 1:** X-ray data collection, structure solution and refinement Values in parentheses are for the outer shell.

	Env-GH191	*Fs*GH114	*Tm*GH191_nat†	*Tm*GH191_soak†
Beamline	I04-1	I04	P11	BL 14.2
Data-collection temperature (K)	100	100	100	100
Wavelength (Å)	0.9119	0.9795	1.003	0.9184
Space group	*C*222_1_	*P*3_1_21	*P*12_1_1	*P*12_1_1
*a*, *b*, *c* (Å)	41.84, 185.32, 128.39	95.57, 95.57, 231.43	195.19, 84.50, 195.48	196.06, 84.50, 196.63
α, β, γ (°)	90, 90, 90	90, 90, 120	90, 119.91, 90	90, 119.83, 90
Total reflections	809771 (40706)	1943673 (94000)	1487233 (75339)	728321 (37378)
Unique reflections	61821 (3055)	158994 (7793)	216213 (10715)	171234 (8538)
Completeness (%)	100.0 (100.0)	100.0 (100.0)	100.0 (100.0)	99.5 (99.8)
Multiplicity	13.1 (13.3)	12.2 (12.1)	6.9 (7.0)	4.3 (4.4)
*R*_meas_ ‡	0.175 (3.466)	0.123 (2.563)	0.210 (2.280)	0.252 (1.452)
*R*_p.i.m._ §	0.067 (1.313)	0.049 (1.027)	0.080 (0.855)	0.121 (0.688)
〈*I*/σ(*I*)〉	11.5 (0.8)	11.2 (1.0)	6.4 (0.9)	5.4 (1.1)
Resolution range (Å)	46.3–1.64 (1.67–1.64)	82.8–1.61 (1.64–1.61)	48.9–2.4 (2.44–2.40)	49.21–2.60 (2.64–2.60)
CC_1/2_¶	0.998 (0.467)	0.999 (0.461)	0.985 (0.412)	0.978 (0.360)
Wilson *B* factor (Å^2^)	14.6	20.4	41.0	31.7
No. of reflections, working set	58581	150799	205033	162629
No. of reflections, test set	3200	8055	11162	8591
Final *R*_cryst_	0.197 (0.340)	0.178 (0.340)	0.237 (0.340)	0.216 (0.340)
Final *R*_free_	0.241 (0.370)	0.195 (0.376)	0.264 (0.360)	0.243 (0.360)
Coordinate error†† (Å)	0.113	0.075	0.172	0.194
Asymmetric unit				
No. of protein molecules	1.33‡‡	3	6	6
No. of non-H protein atoms	4257	6869	29067	29171
No. of non-H solute atoms	475	1043	540	596
R.m.s.d.s				
Bond lengths (Å)	0.0079	0.0102	0.0118	0.0129
Angles (°)	1.45	1.70	2.116	2.356
Average *B* factors (Å^2^)				
Protein	20.2	28.7	*A*, 50.5; *B*, 50.6; *C*, 56.0; *D*, 56.7; *E*, 57.0; *G*, 55.7	*A*, 27.4; *B*, 40.0; *C*, 41.9; *D*, 42.8; *E*, 42.5; *F*, 42.5
Solute	30.2	37.8	50.4	33.9
*MolProbity* score	0.90	0.91	2.1	2.0
Clashscore	0.96	0.44	0.59	0.39
Ramachandran plot				
Most favoured (%)	97.4	96.3	96.2	96.7
Allowed (%)	2.6	3.7	3.8	3.3
Outliers (%)	0.0	0.0	0.0	0.0
Rotamer outliers (%)	0.2	0.3		
PDB code	9ep5	9ep6	9eux	9euz
X-ray images record at Zenodo	https://doi.org/10.5281/zenodo.10936959	https://doi.org/10.5281/zenodo.11011296	https://doi.org/10.5281/zenodo.14389479	N/A
				

† *Tm*GH191 is isomorphous to PDB entry 2aam and has a sequence identity of 100%.

‡ Diederichs & Karplus (1997 ▸).

§ Weiss & Hilgenfeld (1997 ▸).

¶ Karplus & Diederichs (2012 ▸).

†† *R*-factor-based coordinate DPI (equation 26 in Cruickshank, 1999 ▸).

‡‡ One of two protein molecules (2114 non-H atoms) and the associated water molecules and sulfate ion (115) are assigned an occupancy of 0.33.

**Table 2:** Comparison of GH191 and GH114 structures solved in this work Structure superposition was carried out using *SSM* (Krissinel & Henrick, 2004 ▸) as incorporated in *Coot* (Emsley *et al.*, 2010 ▸). Ligand-bound Ega3 and *Tm*GH191 are not included in the comparison.

	Bacterial	Fungal	Cross-species			
	*Tm*GH191/Env-GH191	*Fs*GH114/Ega3	*Tm*GH191/Ega3	Env-GH191/Ega3	*Tm*GH191/*Fs*GH114	Env-GH191/*Fs*GH114
Aligned residues	252	218	187	162	178	190
Sequence identity (%)	28.2	34.9	19.3	17.3	19.7	18.4
R.m.s.d. (Å)	1.48	1.49	1.75	3.01	2.31	2.56
