# Supplementary material for: Structures of α-galactosaminidases from the CAZy GH114 family and homologs defining a new GH191 family of glycosidases
Source: Acta Crystallogr D Struct Biol. 2025 Apr 15;81(Pt 5):234–51. doi: 10.1107/S2059798325002864 (PMC12054363; doi:10.1107/S2059798325002864)
Supplement: Supplementary file 1 [file d-81-00234-sup1.pdf]

STRUCTURAL
BIOLOGY

Volume 81 (2025)

Supporting information for article:

Structures of α-galactosaminidases from the CAZy GH114 family and homologs defining a new GH191 family of glycosidases

Christian Roth, Olga V. Moroz, Suzan A. D. Miranda, Lucas Jahn, Elena V. Blagova, Andrey A. Lebedev, Dorotea R. Segura, Mary A. Stringer, Esben P. Friis, João P. L. Franco Cairo, Gideon J. Davies and Keith S. Wilson

S1. Methods

S1.1. GH191 activity assay by MALDI

An additional Pel substrate degradation assay was performed to further validate enzyme activity and confirm the polymer composition. The solid Pel substrate was diluted to approximately 2 mg/ml in 50 mM ammonium acetate buffer (pH 6.0) supplemented with 1% ethanol and heated at 95°C with shaking for 5 minutes for solubilization purposes. Following this, 1 µl of 15 mg/ml enzyme was added to 100 µl of the diluted substrate solution. The reactions were carried out in three replicates at 30°C with shaking at 750 rpm for 1 hour. After centrifugation of the reaction at top speed for 10 minutes, 1 µL of the reaction supernatant was mixed with 1 µL of MALDI matrix solution (20 mg/mL 2,5 dihydroxybenzoic acid (DHB) dissolved in 30% acetonitrile, 0.1% trifluoracetic acid) and spotted on a Bruker MTP 384 ground steel MALDI plate and dried under a bulb lamp for 20 minutes prior to data collection. Experiments were performed on a Bruker UltrafleXtreme MALDI-TOF/TOF instrument. The data acquired over the range of *m/z* 250 to 3000 were collected in positive-ion mode by averaging 8000 laser shots using the 50% to 100% laser power sufficient to obtain adequate signal-to-noise ratios.

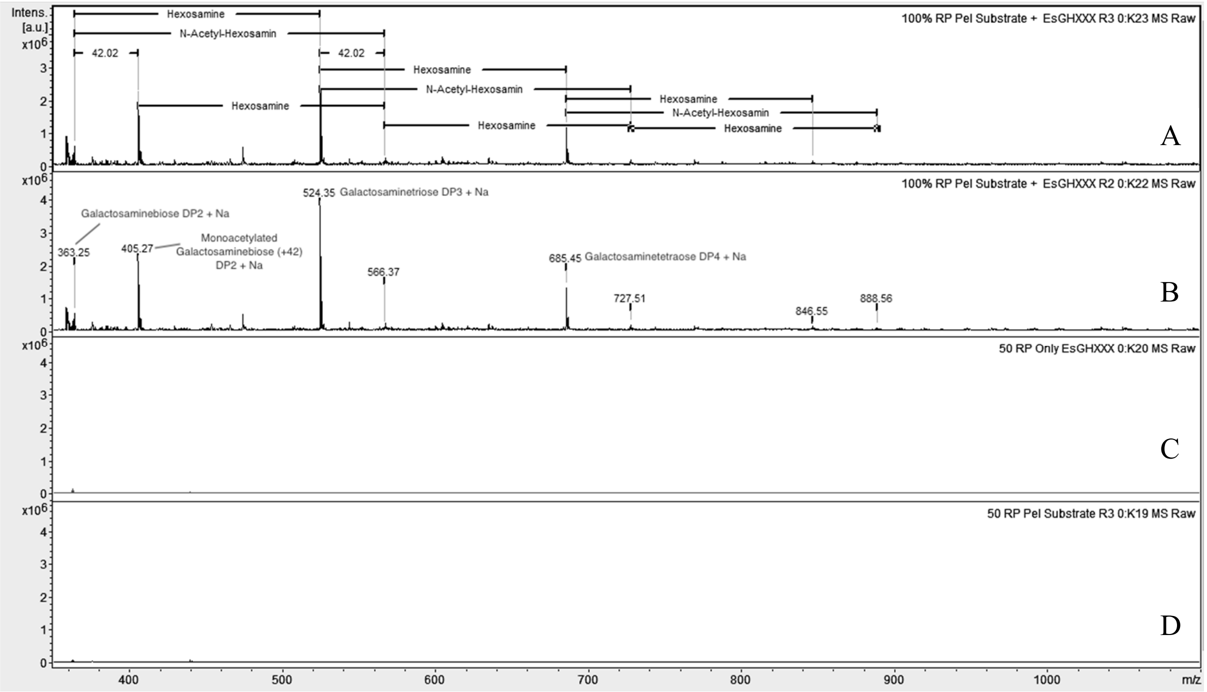

Figure S1 Pel Substrate Degradation. Panels **A** and **B** show the ion products from the enzymatic reaction of Env-GH191 with Pel substrate. The main oligosaccharides generated are observed as sodiated (Na^+) peaks at *m/z* 524 and *m/z* 685 corresponding to a degree of polymerization (DP) of 3 (galactosaminetriose) and 4 (galactosaminetetraose), respectively. Other peaks are relatively minor. The peak at *m/z* 405 has been identified as a monoacetylated galactosamine disaccharide. This peak is +42 Da relative to the low intensity DP2 hexosaminebiose peak at *m/z* 363, indicating that one of the galactosamine units is acetylated while the other is not. If both units were acetylated, the expected *m/z* would be 447 (+84 Da), matching N-Acetyl-glucosamine disaccharide (chitobiose) for the sake of comparison. Panel **C** is the control reaction containing only Env-GH191 and panel **D** is the control reaction containing only the Pel substrate. These findings align with the analysis by Le Mauff *et al.* (2022) https://doi.org/10.1038/s42003-022-03453-2, which proposed that the Pel substrate consists of both α-1,4-linked galactosamine and N-acetylgalactosamine, primarily in the form of dimer repeating units (Le Mauff *et al.*, 2022).

References

Le Mauff, F., Razvi, E., Reichhardt, C., Sivarajah, P., Parsek, M. R., Howell, P. L. & Sheppard, D. C. (2022). *Commun. Biol.* **5**, 502.
